# Assessing field performance of ultrasensitive rapid diagnostic tests for malaria: a systematic review and meta-analysis

**DOI:** 10.1186/s12936-021-03783-2

**Published:** 2021-06-03

**Authors:** Celestin Danwang, Fati Kirakoya-Samadoulougou, Sekou Samadoulougou

**Affiliations:** 1 Department of Surgery and Specialties, Faculty of Medicine and Biomedical Sciences, University of Yaoundé I, Yaoundé, Cameroon; 2 Epidemiology and Biostatistics Unit, Institut de Recherche Expérimentale Et Clinique, Université Catholique de Louvain, Brussels, Belgium; 3 Centre for Research in Epidemiology, Biostatistics and Clinical Research, School of Public Health, Université Libre de Bruxelles (ULB), Route de Lennik, 808, 1070 Bruxelles, Brussels, Belgium; 4 Centre for Research On Planning and Development (CRAD), Laval University, Quebec, G1V 0A6 Canada; 5 Evaluation Platform On Obesity Prevention, Quebec Heart and Lung Institute, Quebec, G1V 4G5 Canada

## Abstract

**Background:**

To overcome the limitations of conventional malaria rapid diagnostic tests (cRDTs) in diagnosing malaria in patients with low parasitaemia, ultrasensitive malaria rapid diagnostic tests (uRDTs) have recently been developed, with promising results under laboratory conditions. The current study is the first meta-analysis comparing the overall sensitivity, and specificity of newly developed ultrasensitive *Plasmodium falciparum* malaria RDT (Alere™ Ultra-sensitive Malaria Ag P. *falciparum* RDT) with the cRDT conducted in the same field conditions.

**Methods:**

PubMed, EMBASE, Cochrane infectious diseases group specialized register, and African Journals Online (AJOL) were searched up to 20^th^ April 2021. Studies with enough data to compute sensitivity and specificity of uRDT and cRDT were retrieved. A random-effect model for meta-analysis was used to obtain the pooled sensitivity and specificity.

**Results:**

Overall, 15 data sets from 14 studies were included in the meta-analysis. The overall sensitivity of the Alere™ ultra-sensitive Malaria Ag *P. falciparum* RDT regardless of the reference test and the clinical presentation of participants, was 55.5% (95% confidence interval [CI]: 45.5; 65.0), while the sensitivity regardless of the reference test and the clinical presentation of participants, was 42.9% (95% CI: 31.5; 55.2) for the cRDT performed in the same field conditions. When PCR was used as reference test, the sensitivity of uRDT was 60.4% (95% CI: 50.8; 69.2), while the sensitivity was 49.4% (95% CI: 38.2; 60.6) for the cRDT. The pooled specificity of uRDT regardless of the reference test and the clinical presentation of participants was 98.6% (95% CI: 97.1; 99.4), and the pooled specificity of cRDT regardless of the reference test and the clinical presentation of participants was 99.3% (95% CI: 98.1; 99.7). When PCR was used as reference test the specificity of uRDT and cRDT was 97.5% (95% CI: 94.1; 98.9) and 98.2% (95% CI: 95.5; 99.3). Regardless of the reference test used, the sensitivity of Alere™ Ultra-sensitive Malaria Ag *P. falciparum* RDT in symptomatic patients was 72.1% (95%CI: 67.4; 76.4), while sensitivity of cRDT was 67.4% (95%CI: 57.6; 75.9).

**Conclusion:**

Findings of the meta-analysis show that Alere™ Ultra-sensitive Malaria Ag *P. falciparum* RDT compared to cRDT performed in the same field conditions has higher sensitivity but lower specificity although the difference is not statistically significant.

**Supplementary Information:**

The online version contains supplementary material available at 10.1186/s12936-021-03783-2.

## Background

Even though the global burden of malaria has been reduced since 2000, in 2018, nearly 228 million new cases of malaria were recorded globally, and there were close to 405.000 excess death caused by malaria [1]. Among those deaths, children [2] and pregnant [3] women represent the most vulnerable population. To reduce and eliminate malaria infections, the World Health Organization (WHO) recommends the use of the « Test, Treat and Track» strategy [4]. The aim of this strategy is to make sure that every suspected case is tested using a confirmation test, and every confirmed case is treated with the appropriate anti-malaria medication [4].

In people living in areas where malaria is prevalent, and in pregnant women, malaria diagnosis can be challenging. In the former, because every suspected case must be diagnosed and treated, even those with parasitaemia below the detection threshold of conventional rapid diagnostic tests (cRDTs) for malaria, and in the latter, because of the ability of *Plasmodium falciparum* to bind to the placenta, which can lead to parasite densities in peripheral blood below the detection threshold of the most used cRDTs and light microscopy, hence the need for ultrasensitive diagnostic tests (uRDTs) [5].

Currently, nucleic acid amplification tests are known to be sensitive to detect these low-density infections [6, 7]. Nonetheless, these methods are limited to well-equipped laboratory settings due to their inherent complexity and need for sophisticated laboratory facilities. Recently, to fulfil the demand for diagnostic tests that are cheaper, faster, with high-sensitivity and deployable in the field, uRDT was developed [8]. Like cRDTs, which detect proteins such as histidine-rich protein 2 (HRP2), aldolase, and parasite lactate dehydrogenase (pLDH), they are based on the immunodetection of HRP2 and exhibit promising results when their performance is assessed in laboratory conditions [8].

However, no study has hitherto evaluated through meta-analysis the performance of uRDT under field conditions. This first systematic review with meta-analysis aimed to compare the overall sensitivity, and specificity of newly developed ultrasensitive malaria RDT (Alere™ Ultra-sensitive Malaria Ag *P. falciparum* RDT) with the cRDT conducted in the same field conditions.

## Methods

The review is conducted in accordance with the recommendations for diagnostic test accuracy meta-analysis in the Cochrane Handbook for Systematic Reviews [9] and is reported with respect to the Preferred Reporting Items for Systematic Review and Meta-analysis of Diagnostic Test Accuracy Studies [10]. The current review is registration with PROSPERO CRD42021227784.

### Search strategy

PubMed, EMBASE, Cochrane infectious diseases group specialized register, and African Journals Online (AJOL) were searched from inception up to 20 April 2021 with the following terms: (“malaria[tiab]” OR “malaria [MESH]”) AND ("ultrasensitive"[tiab] OR "highly sensitive" [tiab] OR "hypersensitive"[tiab] OR "high-sensitive"[tiab] OR "high sensitive"[tiab] OR "RDT"[tiab]). The search strategy used in PubMed which was adapted to fit with other databases is presented in Additional file 1: Table S1.

After bibliographic search, the titles and abstracts were screened for eligibility and duplicates were removed. Full texts of potentially eligible articles were retrieved and assessed for final inclusion independently by two reviewers, with discrepancy between both resolved by discussion.

### Eligibility criteria

Both observational and experimental studies reporting enough data to compute sensitivity and specificity of uRDT and cRDT in the same setting and comparing both with the same reference test were included in the meta-analysis. Only studies conducted on the field (not in the laboratory), regardless of the language and year of publication were retained. We excluded editorials, reviews, letters, commentaries, and studies lacking key data.

### Quality assessment

The Quality Assessment of Diagnostic Accuracy Studies 2 (QUADAS-2) was used independently by two reviewers to assess the quality of included studies [11]. Disagreement between the two reviewers were resolved by discussion.

### Data extraction

The following information was retrieved on a preconceived data extraction form by one reviewer: the name of the first author, the country where the study was conducted, the year of publication, the characteristics of the study population in terms of symptoms and age, the presence of pregnant women in the sample, the commercial name of the uRDT and reference test used in the study, the storage condition of the uRDT, the number of true positive (TP), true negative (TN), false positive (TP), false negative (FN).

Articles reporting on diagnostic performance of uRDT in different age categories (e.g., adults and children) or in which uRDT was compared with more than one reference test were divided in separated data sets. Thereafter, a second reviewer checked the concordance between data extracted and the content of the article prior to the data synthesis and analysis.

### Statistical analysis

The “meta” package within R software version 4.0.2 was used for analysis [12, 13]. A random-effect model was used to obtain the overall summary effect of studies reporting enough data to compute the sensitivity, and specificity. The Clopper-Pearson method was used to compute the confidence intervals and the maximum-likelihood estimator was used to estimate the between-study variance. The QUADAS-2 score was used to estimate the risk of bias in included studies. A P-value of 0.05 was considered statistically significant in all the analysis.

## Results

### Search results

**Fig. 1:**
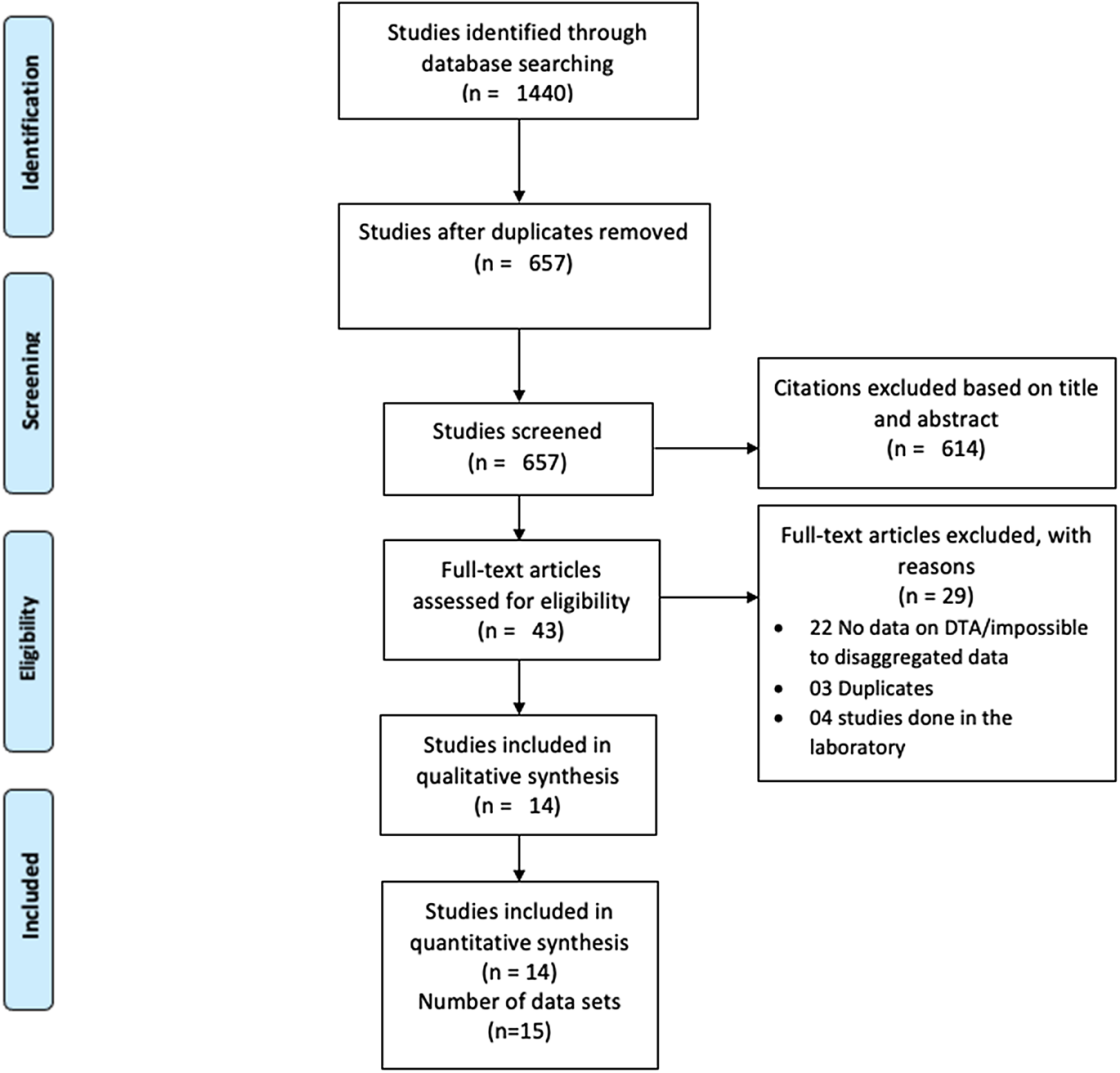
The PRISMA flow diagram

The bibliographic search yielded 1440 articles. The screening based on title and abstracts and full text allowed to retain 15 data sets from 14 studies for the quantitative synthesis as depicted in the Prisma flow diagram (Fig. 1).

### Characteristics of the included studies

Studies were published between 2018 and 2021, with 9 out of 14 conducted in sub-Saharan African countries. Two out of the 14 studies were from Myanmar [14, 15], two from Tanzania [16, 17], while Benin [18], Cambodia [19], Ethiopia [20], Indonesia [21], Kenya [22], Mozambique [23], Papua New Guinea [24], Ghana [25], Colombia [26], and The Gambia [27] were represented by one study (Table 1).

**Table 1:** Characteristics of the studies included in the review

Author	Year	Design	Country	Setting	Mean* age	Minimum age	Maximum age	%Male	Characteristic of the population	Plasmodium *sp* target by the Ultrasensitive RDT	Person(s) performing ultrasensitive RDT	Characteristics of patients in terms of symptoms	Reference test	Commercial name(s) of the ultra-sensitive RDT used in the study	Parasite density reported in the study
Briand	2020	Cross sectional analysis of a cohort study	Benin	Hospital based	26.7	NR	NR	NA	Pregnant women	Falciparum	Unclear	Asymptomatic and symptomatic	PCR	Alere™ Ultra-sensitive Malaria Ag P. falciparum RDT	Mean parasite density (95%CI): 20.7 p/μL (10.8–39.6)
Yeung	2020	Cross sectional	Cambodia	Population- based, Hospital based	NR	NR	NR	NR	All age categories and conditions (Active case detection)	Falciparum	Trained lab technician	Asymptomatic and symptomatic	PCR	Alere™ Ultra-sensitive Malaria Ag P. falciparum RDT	Unclear
Girma	2019	Cross sectional	Ethiopia	Population-based	NR	NR	NR	NR	All age categories and conditions	Falciparum	Trained lab technician	Asymptomatic	Ultra-sensitive PCR	Alere™ Ultra-sensitive Malaria Ag P. falciparum RDT	uRDT median = 7817 per mL, and cRDT median = 70,205 (IQR: 5585–196 950 per mL)
Unwin	2020	Cross sectional	Indonesia	Hospital based	NR	NR	NR	NA	Pregnant women	Falciparum	NR	Asymptomatic	Composite test (qPCR, LAMP, nPCR)	Alere™ Ultra-sensitive Malaria Ag* P. falciparum* RDT	Unclear
Samuels	2019	Cross sectional analysis of a cohort study	Kenya	Population-based	NR	NR	NR	NA	Pregnant women	Falciparum	Unclear	Asymptomatic and symptomatic	PCR	Alere™ Ultra-sensitive Malaria Ag* P. falciparum* RDT	Median = 148 (IQR: 11–1260 per μL)
Galatas	2020	Cross sectional	Mozambique	Population-based	4	NR	NR	44.8	All age categories and conditions	Falciparum	Trained lab technician	Asymptomatic and symptomatic	PCR	Alere™ Ultra-sensitive Malaria Ag* P. falciparum* RDT	Geo mean = 112.9 p/μL. (Ranged: 0.6 to 120,786.2) for uRDT, And Geo mean = 145.5 p/μL. (Ranged: 0.6–120,786.2) for cRDT positive samples
Landier_1	2018	Cross sectional	Myanmar	Population-based	36	18	NR	47.1	Adults	Falciparum	NR	Asymptomatic	ELISA	Alere™ Ultra-sensitive Malaria Ag* P. falciparum* RDT	Geo mean uRDT + : 3,019 p/ml (95% CI, 1,790 to 5,094 p/ml), and cRDT + : 11,352 (95% CI, 5,643 to 22,837 p/ml)
Landier_2	2018	Cross sectional	Myanmar	Population-based	36	18	NR	47.1	Adults	Falciparum	NR	Asymptomatic	Ultra-sensitive PCR	Alere™ Ultra-sensitive Malaria Ag P. falciparum RDT	Geo mean uRDT + : 3,019 p/ml (95% CI, 1,790 to 5,094 p/ml), and cRDT + : 11,352 (95% CI, 5,643 to 22,837 p/ml)
Liu	2019	Cross sectional	Myanmar	Population-based	14	0.6	90	43.1	All age categories and conditions	Falciparum	Unclear	Asymptomatic and symptomatic	PCR	Alere™ Ultra-sensitive Malaria Ag* P. falciparum* RDT	NR
Hofmann	2018	Cross sectional	Papua New Guinea	Population-based	29	NR	NR	NR	5 years and older (excluding pregnant women)	Falciparum	NR	Asymptomatic and symptomatic	PCR	Alere™ Ultra-sensitive Malaria Ag P. falciparum RDT	Geo mean uRDT + : 427.58 p/μL; median, 173.63 p/μL (IQR, 26.89–547.75 p/μL), and Geo mean cRDT + : 6104.25 p/μL; median, 1309.40 p/μL (IQR, 135.3–12,586.2 p/μL)
Hofmann_1	2019	Cross sectional analysis of a cohort study	Tanzania	Hospital based	NR	0.2	4.9	NR	Children under 5 year	Falciparum	Unclear	Symptomatic	Ultra-sensitive PCR	Alere™ Ultra-sensitive Malaria Ag P. falciparum RDT	Geo mean:3844 p/μL; median, 54,742 p/μL (IQR, 13–385,514 p/μL)
Hofmann_2	2019	Cross sectional analysis of a cohort study	Tanzania	Hospital based	NR	18	80	NR	Adults	Falciparum	Unclear	Symptomatic	Ultra-sensitive PCR	Alere™ Ultra-sensitive Malaria Ag* P. falciparum* RDT	Geo mean: 1102 p/μL; median, 1691 p/μL (IQR, 27–87,812 p/μL)
Mwesigwa	2019	Cross sectional	The Gambia	Population-based	13	NR	NR	44.3	All age categories and conditions	Falciparum	Nurse	Asymptomatic and symptomatic	PCR	Unclear	NR
Vásquez	2020	Cross sectional	Colombia	Hospital based	NR	NR	NR	NA	Pregnant women	Falciparum	Trained lab technician	Asymptomatic and symptomatic	PCR	Alere™ Ultra-sensitive Malaria Ag* P. falciparum* RDT	Geo mean = 13.2 p/μL (Ranged: 0.03 to 8145)
Manjurano	2021	Cross sectional	Tanzania	Hospital based	NR	5	NR	42.8	5 years and older	Falciparum	Trained lab technician	Symptomatic	PCR	Alere™ Ultra-sensitive Malaria Ag* P. falciparum* RDT	Ranged: 40–1,000,000 p/μL
Acquah	2021	Cross sectional	Ghana	Population-based	NR	3	88	33	All age categories and conditions	Falciparum	Trained lab technician	Asymptomatic	PCR	Alere™ Ultra-sensitive Malaria Ag* P. falciparum* RDT	Unclear

*Mean or median age

**Fig. 2:**
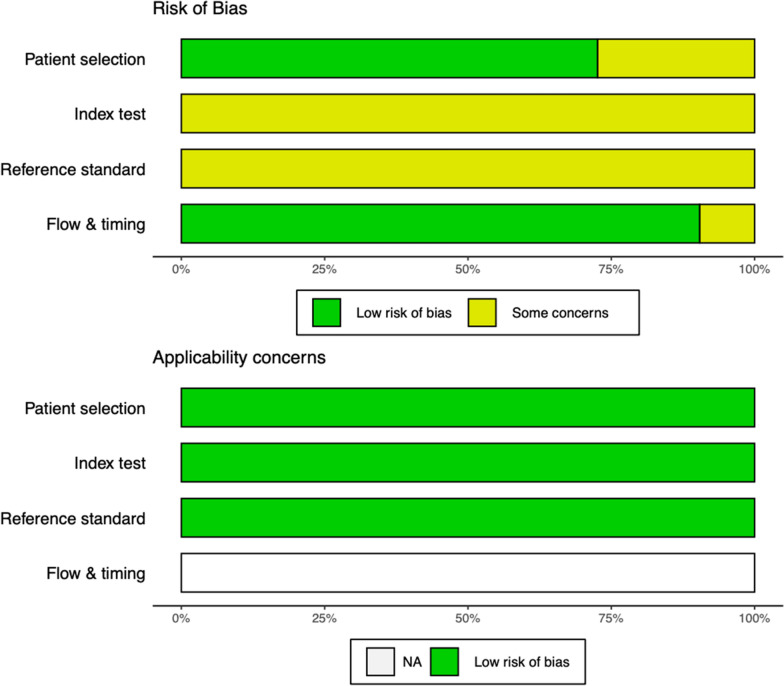
Quality assessment of studies included in the meta-analysis

Eight studies (57.1%) were conducted in patients regardless of the presence of symptoms, while two (14.3%) were conducted in symptomatic patients and four (28.6%) in asymptomatic patients. The mean/median age of the study population ranged from 4 to 36 years as reported by seven studies. Four (28.6%) studies were conducted in pregnant women (Table 1). *Plasmodium falciparum* was the species targeted by the uRDT test in all the studies, and all the uRDT were from the same manufacturer (abbott Alere™ Ultra-sensitive Malaria Ag *P. falciparum* RDT). The reference test was mainly PCR (10 data sets), and ultrasensitive PCR (4 data sets) (Table 1). The risk of bias in studies included in the review ranged from low to moderate and is summarized in Fig. 2.

### Meta-analysis of sensitivity and specificity

#### Sensitivity

**Fig. 3:**
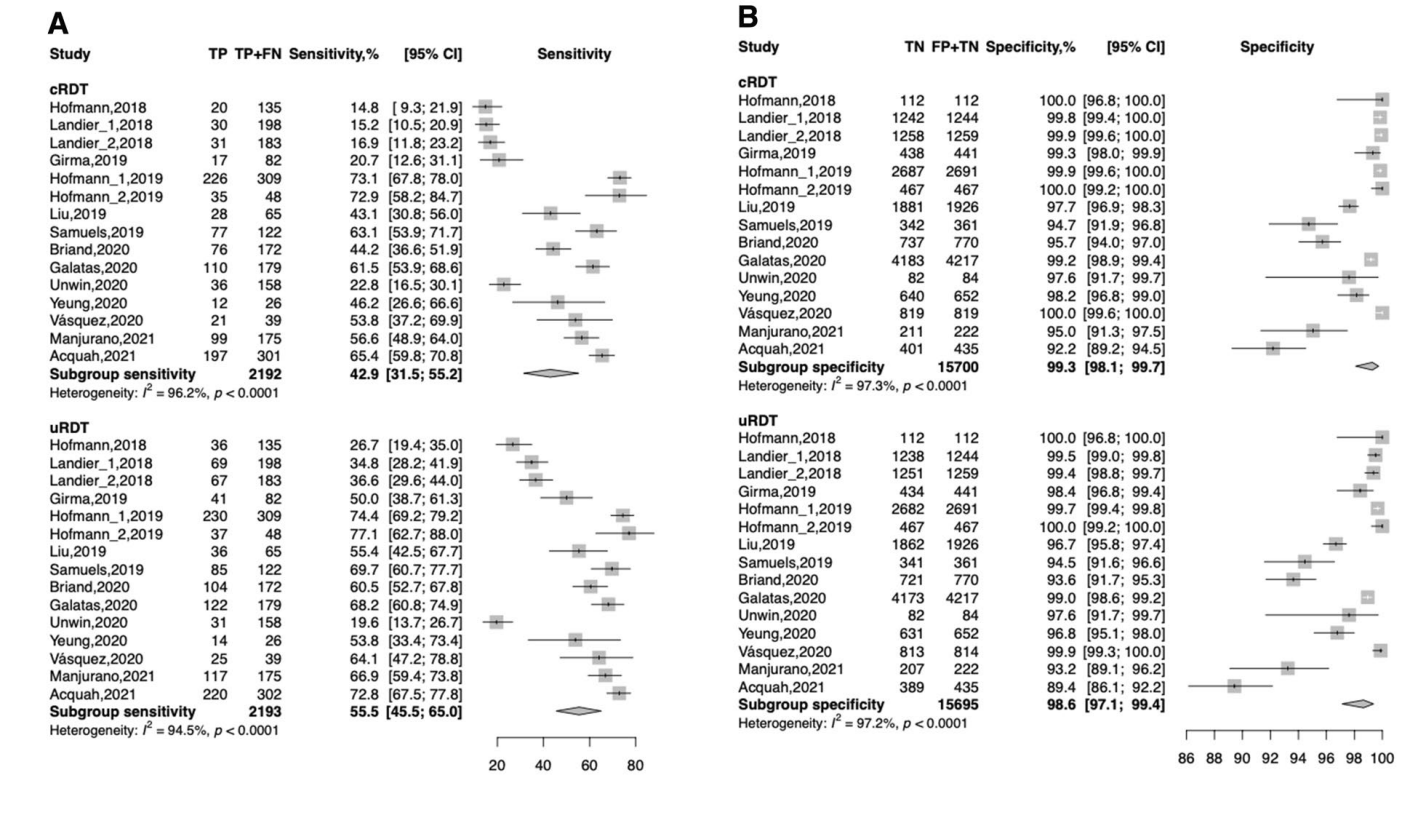
The forest plot of sensitivity and specificity

Even if the difference was not statistically significant, the Alere™ Ultra-sensitive Malaria Ag *P. falciparum* RDT had a higher sensitivity than the cRDT performed in the same field conditions. The overall sensitivity of the uRDT was 55.5% (95% CI: 45.5; 65.0) while the figure was 42.9% (95% CI: 31.5; 55.2) for the cRDT (Fig. 3). The difference in terms of sensitivity between the Alere™ Ultra-sensitive Malaria Ag *P. falciparum* RDT and cRDT varies according to the reference test used. When PCR was used as reference test, the sensitivity of Alere™ Ultra-sensitive Malaria Ag *P. falciparum* RDT was 60.4% (95% CI: 50.8; 69.2) while it was 49.4% (95% CI: 38.2; 60.6) for the cRDT. When the ultra-sensitive PCR was used as reference test, sensitivity of uRDT was 60.3% (95% CI: 42.2; 75.9) and of cRDT was 44.1% (95% CI: 18.8; 72.8) (Table 2).

**Table 2:** Meta-analysis of diagnostic performance Alere™ Ultra-sensitive Malaria Ag P. falciparum RDT according to the reference test

N data sets		Pooled sensitivity, % [95% CI]		Heterogeneity for sensitivity (I^2^, %)		Pooled specificity, % [95% CI]		Heterogeneity for specificity (I^2^, %)	
		uRDT	cRDT	uRDT	cRDT	uRDT	cRDT	uRDT	**cRDT**
According to reference test									
PCR	9	60.4 [50.8; 69.2]	49.4 [38.2; 60.6]	89.4	92.6	97.5 [94.1; 98.9]	98.2 [95.5; 99.3]	97.6	97.1
Ultra-sensitive PCR	4	60.3 [42.2; 75.9]	44.1 [18.8; 72.8]	93.6	97.1	99.5 [98.9; 99.8]	99.8 [99.7; 99.9]	71.9	0.0

*PCR* Polymerase chain reaction, *RDT* Malaria rapid diagnostic test, *CI* Confidence interval

The sensitivity of Alere™ Ultra-sensitive Malaria Ag *P. falciparum* RDT and cRDT in pregnant women was 52.5% (95% CI: 31.3; 72.9) and 44.9% (95% CI:29.7; 61.2) respectively. Regardless of the reference test used, the sensitivity of Alere™ Ultra-sensitive Malaria Ag *P. falciparum* RDT in symptomatic patients was 72.1% (95%CI: 67.4; 76.4), while sensitivity of cRDT was 67.4% (95%CI: 57.6; 75.9). In asymptomatic patients the sensitivity of Alere™ Ultra-sensitive Malaria Ag *P. falciparum* RDT was 42.1% (95%CI: 26.5; 59.5), and sensitivity of cRDT was 26.0% (95%CI: 13.9; 43.4).

#### Specificity

The overall specificity of the Alere™ Ultra-sensitive Malaria Ag *P. falciparum* RDT was lower than the cRDT (statistically non-significant). The pooled specificity of Alere™ Ultra-sensitive Malaria Ag *P. falciparum* RDT was 98.6% (95% CI: 97.1; 99.4), and of cRDT was 99.3% (95% CI: 98.1; 99.7). When PCR was used as reference test the specificity of uRDT and cRDT was 97.5% (95% CI: 94.1; 98.9) and 98.2% (95% CI: 95.5; 99.3) respectively, and when the ultra-sensitive PCR was used as reference test the specificity was 99.5% (95%CI: 98.9; 99.8) and 99.8% (95% CI: 99.7; 99.9), respectively (Table 2).

The specificity of Alere™ Ultra-sensitive Malaria Ag *P. falciparum* RDT and cRDT in pregnant women was 98.1% (95% CI: 91.5; 99.6) and 98.7% (95% CI: 90.9; 99.8), respectively. Regardless of the reference test used, the specificity of Alere™ Ultra-sensitive Malaria Ag *P. falciparum* RDT in symptomatic patients was 99.5% (95%CI: 92.6; 100.0), and specificity of cRDT was 99.7% (95%CI:95.0; 100.0). In asymptomatic patients the specificity of Alere™ Ultra-sensitive Malaria Ag *P. falciparum* RDT was 98.4% (95%CI: 95.4; 99.5), and specificity of cRDT was 99.3% (95%CI: 96.8; 99.9).

## Discussion

This meta-analysis assessing the field performance of malaria uRDT (Alere™ Ultra-sensitive Malaria Ag *P. falciparum* RDT) highlights the higher sensitivity of the Alere™ Ultra-sensitive Malaria Ag *P. falciparum* RDT compared to the cRDT when performed on the same field conditions and confirmed findings observed in the laboratory conditions [8]. Indeed, these results are promising for detection of malaria in patients with low parasitaemia, subclinical or asymptomatic infections and pregnant women. For the former because current cRDT available on the market and widely used in malaria endemic countries are not able to diagnose malaria in patients with a parasite density below 100 /μL while uRDT can, this may explain the relatively higher sensitivity of uRDT [28]. For the latter because they are in most countries under intermittent preventive treatment (IPT), which can strongly influence the parasite density, and because of pathophysiology of malaria during pregnancy.

Indeed, in pregnant women, red blood cells parasite by *Plasmodium* bind to the chondroitin sulfate portion of syndecan‐1 of both intervillous space and the syncytiotrophoblast [5] leading to their sequestration into the placenta and explain a relative low blood parasite density [29, 30], and thus their capacity to escape to cRDT. Malaria in pregnancy is deleterious for both the mother [29] and the fetus [1]. For the mother, malaria can cause anaemia, severe disease, and death while for the fetus and newborn it contributes to stillbirth, preterm birth, and low birthweight [1, 5]. The WHO estimated that 822,018 of cases of low birthweights in sub-Saharan Africa were related to exposure to malaria parasite during pregnancy in 2019. Given the tremendous burden of malaria in pregnant women, there is an urgent need of highly sensitive method that can help in timely efficient diagnosing of malaria in this vulnerable population. Interestingly the current meta-analysis found that uRDT performed better than cRDT in this specific population, which may allow to capture and treat additional cases that may have been missed by cRDT.

Even if the specificity of uRDT seems to be slightly lower than cRDT it is estimated to 98.6% (95% CI: 97.1; 99.4) and is higher than 95% regardless of the reference test used in blood. Importantly its specificity is not statistically significantly different from the one obtained for the cRDT in the current meta-analysis.

From a public health perspective, the findings of this study suggest that Alere™ Ultra-sensitive malaria Ag *P. falciparum* RDT is more sensitive than cRDT and could help to capture additional low parasite density malaria cases that escape the current cRDT. These results call for the assessment of additional criteria, namely the stability at high temperature, cost and shelf life of uRDTs, before Alere™ Ultra-sensitive malaria Ag *P. falciparum* RDT could be integrated into the already available malaria diagnostic arsenal. Furthermore, it is essential that a correlation be established in the field between the parasitaemia observed in patients and the positivity of uRDTs to confirm the results obtained in the laboratory conditions. Nevertheless, given that cRDTs typically do not consistently detect parasite densities lower than 100p/µL, the definition of an ultrasensitive malaria test needs to be clarified and the conditions to fulfil for a test to be considered ultrasensitive need to be consensually adopted to compliment the current WHO definition, which is based solely on the parasite density detection threshold (below 100 parasites/μl) [28].

The results of the current study must be interpreted considering some drawbacks. Most of the studies were conducted in WHO African region (sub-Saharan Africa), which is the region with the highest burden of malaria. This can limit the generalizability of the results to other malaria endemic regions of the world. Data does not allow for stratified analysis according to parasite density, which is one of the key elements in the performance of malaria diagnostic tests. None of the studies specified the storage conditions of uRDT and cRDT, and only seven reported clearly that the test was conducted by trained laboratory technician/nurse, this may have impacted on the quality of the results. Several brands of cRDT with different performances were used as comparators to the Alere™ Ultra-sensitive Malaria Ag P. falciparum RDT in the current study. The diversity of these tests could be an additional source of heterogeneity in the results and may limit a direct comparison between Alere™ Ultra-sensitive Malaria Ag *P. falciparum* RDT and a specific brand of cRDT test. Nevertheless, this study is the first to assess by the mean of a meta-analysis, the performances of one of the most recent diagnostic tools of malaria diagnostic in the field conditions. Furthermore, recent guidelines were used for the assessment of quality of included studies and the reporting of the review.

## Conclusion

Findings of the meta-analysis show that Alere™ Ultra-sensitive Malaria Ag *P. falciparum* RDT compared to cRDT performed in the same field conditions has higher sensitivity but lower specificity although the difference is not statistically significant.

## Supplementary Information

**Additional file 1: Table S1.** Search strategy for PubMed.

## Data Availability

All materials are available in the manuscript and additional file.
