# Supplementary material for: Assessing field performance of ultrasensitive rapid diagnostic tests for malaria: a systematic review and meta-analysis
Source: Malar J. 2021 Jun 3;20:245. doi: 10.1186/s12936-021-03783-2 (PMC8176703; doi:10.1186/s12936-021-03783-2)
Supplement: Supplementary file 1 — Additional file 1: Table S1. Search strategy for PubMed. [file 12936_2021_3783_MOESM1_ESM.pdf]

Field performances of malaria ultrasensitive rapid diagnostic tests: a systematic review and meta-analysis

Additional file

Additional Tables

Additional file 1: Table S 1. Search strategy for PubMed..2

Additional file 1: Table S 1. Search strategy for PubMed

Search	Search terms
#1	"Malaria"[tiab] OR " Malaria"[MESH]
#2	"ultrasensitive"[tiab] OR "highly sensitive" [tiab] OR "hypersensitive"[tiab] OR "high-sensitive"[tiab] OR "high sensitive"[tiab] OR "RDT"
#3	#1 AND #2
